# Detection and Control of Prion Diseases in Food Animals

**DOI:** 10.5402/2012/254739

**Published:** 2012-02-29

**Authors:** Peter Hedlin, Ryan Taschuk, Andrew Potter, Philip Griebel, Scott Napper

**Affiliations:** ^1^Department of Biochemistry, University of Saskatchewan, Saskatoon, SK, Canada S7N 5E3; ^2^Vaccine and Infectious Disease Organization, University of Saskatchewan, Saskatoon, SK, Canada S7N 5E3; ^3^School of Public Health, University of Saskatchewan, Saskatoon, SK, Canada S7N 5E3

## Abstract

Transmissible spongiform encephalopathies (TSEs), or prion diseases, represent a unique form of infectious disease based on misfolding of a self-protein (PrP^C^) into a pathological, infectious conformation (PrP^Sc^). Prion diseases of food animals gained notoriety during the bovine spongiform encephalopathy (BSE) outbreak of the 1980s. In particular, disease transmission to humans, to the generation of a fatal, untreatable disease, elevated the perspective on livestock prion diseases from food production to food safety. While the immediate threat posed by BSE has been successfully addressed through surveillance and improved management practices, another prion disease is rapidly spreading. Chronic wasting disease (CWD), a prion disease of cervids, has been confirmed in wild and captive populations with devastating impact on the farmed cervid industries. Furthermore, the unabated spread of this disease through wild populations threatens a natural resource that is a source of considerable economic benefit and national pride. In a worst-case scenario, CWD may represent a zoonotic threat either through direct transmission via consumption of infected cervids or through a secondary food animal, such as cattle. This has energized efforts to understand prion diseases as well as to develop tools for disease detection, prevention, and management. Progress in each of these areas is discussed.

## 1. Introduction

Traditionally infectious diseases were limited to the categories of bacterial, viral, fungal, and parasitic. Despite their differences, these agents are all unified by the requirement of genetic material for protein expression and replication of the agent. More recently, however, a novel infectious disease, which appeared to lack its own genome and is capable of causing devastating pathology in humans and animals, was identified. The causative agent of these diseases has been termed a proteinaceous infectious agent or “prion.” Eventually, after much resistance from the scientific community, it was established that a new class of infectious disease had been discovered.

## 2. History

While descriptions of prion diseases in sheep can be traced back hundreds of years, many of the pivotal experiments which defined the unique nature of the prion diseases did not occur until the 1950s. Specifically, many of the pivotal discoveries of prion diseases reflected efforts of anthropologists, pathologists, and physicians in Papua New Guinea where a novel form of neurological disease called kuru was described. Initially attributed to a “slow virus,” further research in the 1970s revealed that this new agent lacked the qualifying features (nucleic acid) necessary for its classification as a virus. The discovery of kuru stimulated research which led to the identification of a broader group of neurodegenerative disorders which were collectively referred to as transmissible spongiform encephalopathies (TSEs). Interestingly, the concept of slow viruses was first described by Sigurdsson while researching neurodegenerative disease in sheep [1], and later, in 1959, a veterinary pathologist hypothesized that kuru and this neurodegenerative disease of sheep, scrapie, were related disorders. At the same time, similar assumptions about kuru and another human neurodegenerative disease called Creutzfeldt-Jacob disease were made [2]. Ultimately work by Gajdusek's group, in which they successfully transmitted kuru to chimpanzees, changed the concepts regarding the cause of neurodegenerative disorders by indicating that a transmissible agent was responsible for the disease [3, 4].

## 3. Prion Diseases

Prion diseases have been shown to occur in a number of different species. Different names were attributed to each of the diseases based on the affected species: scrapie in sheep, Creutzfeldt-Jacob disease (CJD) in humans, bovine spongiform encephalopathy (BSE) in cattle, and chronic wasting disease (CWD) in cervids. While these diseases all seem to share a common mechanism, they display a number of species-specific differences in symptoms, pathology, and transmissibility (Table 1).

In sheep and cattle, the symptoms of prion disease are quite similar. The symptomatic phase of the disease initially presents with rubbing of the head, flanks, and buttocks, a symptom which may last well into the later stages of the disease. Also evident at the early stages is the nibbling of hair on the lower legs. General weakness follows which progresses to the inability to rise. Wasting symptoms appear, such as a loss in body weight despite normal food and water intake. Neurological motor deficits affecting gait include wide-based stance of hind limbs and high-stepping motions in the front limbs, which in severe cases makes the animals appear intoxicated. Further neurological changes include body tremors and personality/behavioural alterations which may manifest as uncooperativeness, excitability, and extreme distress. Once an animal is symptomatic, scrapie and BSE are slowly progressive and lethal within 2 to 6 months [5, 6].

Symptoms of CWD are more subtle in the early stages of disease. They include weight loss, excessive drinking, and urination as well as regurgitation. The motor deficits common to scrapie and BSE are often absent in CWD cases [7]. Despite the lack of easily observable symptoms, histopathology reveals neurological lesions similar to scrapie, BSE, and human prion diseases [8]. It takes anywhere from several weeks to many months for the infected animal to succumb to CWD once it has spread to the nervous system. Some of the more notable similarities and differences between scrapie, BSE, and CWD are presented in (Table 1).

## 4. Scrapie

Scrapie is almost certainly an ancient disease. Historical descriptions of scrapie can be conclusively traced back to 1732, while other sources claim there are descriptions of scrapie as early as the Roman era [9]. The term “scrapie” was first seen in a paper from 1750, but a more common term for it at the time was “distemper” [10]. The majority of written accounts were not produced by scientists or veterinarians, but by farmers, shepherds, and government employees. These individuals were immediately involved with the infected animals and sometimes went to great efforts to cover up the outbreaks because of the associated monetary/economic implications [9]. Similar to current management strategies, acknowledgment of a scrapie-infected animal meant the entire herd would be culled and pasture land quarantined indefinitely [11]. This behaviour indicated a basic understanding of scrapie as an infectious entity.

While historical descriptions of scrapie symptoms were accurate, the understanding of what caused the disease varied greatly. The range of hypotheses of causative agents include thunder, extreme atmospheric conditions, food/nutritional insufficiency, parasites, humidity in the sheep pen, tail docking, mating age, cross-breeding, and inbreeding [9]. The method of transmission was equally debated with ideas ranging from sexual transmission [10], sporadic occurrence, and genetic inheritance. In 1936, after 100 years of failed attempts to experimentally demonstrate scrapie transmissibility, two French scientists successfully infected sheep and goats intracerebrally, intraocularly, epidurally, and subcutaneously using brain and spinal cord suspensions from scrapie-infected sheep [12]. Their experiment confirmed a number of facts that scrapie was infectious, that infection required a long incubation period (18 months), and that the incubation period was affected by species. The infected sheep demonstrated a quicker symptom onset (11–22 months) than goats (25-26 months) [13].

Even within species, sheep possess a unique attribute with regards to TSE transmission. Their susceptibility is influenced by polymorphisms at three different codons (136, 154, and 171) within the PrP sequence [14]. It has been observed that susceptibility to scrapie strongly correlates with valine (V) at position 136, arginine (R) at 154, and glutamine (Q) at 171 (VRQ) [15], whereas resistance is attributed to alanine (A) at 136, with R at 154 and 171 (ARR) [16]. Prion disease is rare in heterozygous ARR animals and virtually undetected in animals homozygous for ARR [17]. These varying susceptibilities to scrapie infection led to a breeding strategy in Europe where sheep flocks were bred to attain the ARR genotype in an attempt to eradicate the transmission of scrapie among herds [18]. Concerns have been raised, however, that sheep selected from these breeding programs may have greater susceptibility to nontraditional forms of scrapie. Specifically atypical forms of scrapie have been reported in resistant sheep, and a novel form of scrapie, Nor98, primarily affects sheep resistant to conventional scrapie [19].

## 5. Chronic Wasting Disease

CWD was first identified at a research station in Fort Collins, Colorado in 1967 [20]. It was not until 1978, however, that the relationship between CWD and other TSEs was recognized following a comparative analysis of neuronal lesions [21] and the accumulation of PrP aggregates was observed [22]. During the 1970s and 80s, CWD existed primarily in the Colorado Rocky Mountains and an area extending along the river valleys draining into Wyoming [23]. However, in 1996, the disease was also detected on an elk farm in the Canadian prairies [7]. To date, CWD has been detected in wild cervids in 17 US states and 2 Canadian provinces. CWD is now known to be one of the most contagious TSEs and can reach a prevalence of 30% in wild populations and as high as 100% in captive cervids [20].

Strategies for containing CWD are extremely problematic because the afflicted animals primarily consist of wild, free-ranging populations. Other complicating factors include the scavenging of contaminated deer carcases by predators ranging from mountain lions to vultures, persistence of CWD prion protein in soil, and a poor understanding of the natural routes of disease transmission [7]. Although oral cross-species transmission to humans has not been directly observed, it was suspected in a few cases of human CJD that the disease may have contracted through eating CWD-infected deer meat [24].

There is also considerable concern that environmental contamination through the increasing prevalence of CWD in wild cervid populations may serve as a reservoir for CWD transmission to farmed deer and cattle. The close ecological and phylogenetic relationship of elk and deer to cattle supports the potential for eventual disease transmission [7]. While the BSE outbreak of the 1980s was successfully addressed through improved feeding practices, it would be more difficult to manage sources of infection from wild animals and/or environmental contamination. It is reassuring that cattle orally inoculated with CWD-infected brains, as well as cattle housed with CWD-positive deer, do not contract a TSE [8]. That cattle inoculated intracerebrally with CWD material do develop a TSE [9, 10] indicates the potential, given the right circumstances, for CWD to overcome this species barrier. Additionally, in the wild, there may be a greater opportunity to overcome this species barrier through intermediate species linking cervids to cattle. While it is difficult to predict the precise nature of the disease that would occur in cattle should CWD transmit from cervids to cattle, the very perception of the disease would be certain to have devastating consequences to the livestock industry.

## 6. Bovine Spongiform Encephalopathy

Since the first BSE diagnosis in 1986 more than 179,000 positive cases have been identified in Great Britain [25] triggering the culling of 750,000 animals [26, 27]. Experts suggested that as many as 3 million BSE-infected animals entered the human and animal food chains undetected [26]. While the recycling of BSE-infected material through cattle feed was likely the primary factor in disease amplification, the source of the original BSE agent remains unknown [28]. It has been suggested that BSE developed spontaneously in cattle via a somatic or germ-line mutation of PrP or that cattle may have been infected by another species such as sheep [29]. Twenty years of rigorous surveillance following the first BSE outbreak have indicated that disease transmission is under control in most of Europe [30]. Mass screening of bovine tissues as part of the surveillance initiatives has identified two new BSE strains, L-Type and H-Type, which are distinct from the classic BSE [31]. These strains are more rare than classic BSE and are usually detected in older animals, thus representing a possible sporadic form of TSE in cattle [32]. Both strains are infectious, maintain their original molecular phenotype upon transmission, and cause distinct neuropathology. In mouse infection studies, the L-type has been observed to be more virulent, replicating faster than both classical BSE and the H-type strain [31].

Discovering the link between BSE and the vCJD outbreak of 1996 brought TSEs to the attention of the public and health professionals. While confirmed cases of vCJD are only at 188 worldwide [49], the potential spread via contaminated blood or tissue products taken from asymptomatic carriers is of great concern.

## 7. Creutzfeldt-Jacob Disease

Prion diseases of livestock animals are the priority of this report. However, given the demonstrated zoonotic potential of BSE, and the concern that CWD may also represent a zoonotic disease, a brief overview of human prion diseases is appropriate to provide context of the different attributes of the various prion diseases. In the 1950s, an epidemic spreading amongst the Fore people of Papua New Guinea gained the attention of health officials. At the peak of the epidemic, as many as 10% of the local Fore population was infected [50]. The disease appeared to affect the central nervous system and displayed symptoms such as progressive ataxia or loss of motor coordination [51]. Derived from the appearance of these symptoms, the disease was given the name kuru, which in the Fore language means “to shiver” [52]. William Hadlow, a veterinary pathologist specialising in TSEs, made the connection between scrapie and kuru in 1959. He noted that epidemiologically, clinically, and pathologically, the two diseases were remarkably similar [53]. This led to further investigations by Gajdusek who was able to demonstrate that kuru was transmissible by successfully infecting chimpanzees [3].

Epidemiological studies suggested that kuru was transmitted by the cannibalistic Fore funeral rituals [54]. These ceremonies involved the children and women consuming nervous tissue from the deceased relative [55]. The number of kuru cases declined steadily following the discontinuation of the funeral ceremonies. Complete eradication of kuru has taken much longer than anticipated due incubation periods lasting as long as four decades [52].

 Currently, the most common form of prion disease in humans is sporadic CJD (sCJD) with a frequency of approximately 0.5–1 case per million people [56]. Unlike the familial forms of CJD, which are caused by autosomal dominant traits linked to mutations on the *Prnp* gene [57], it is unknown whether sCJD is caused by endogenous or exogenous factors [58]. Countries with active surveillance programs, such as Switzerland, often report higher CJD incidence rates with 3 cases per million people [59]. Most of those cases are, however, due to iatrogenic spread rather than ingestion of infected material [60]. Symptoms in sporadic cases usually appear around the age of seventy and are lethal within weeks or months. Symptoms often include rapidly progressing dementia, ataxia, muscle twitching, and uncoordinated speech [56].

 Following the BSE crisis in the United Kingdom in the 1980s, a novel form of CJD emerged. Given the name “variant CJD” (vCJD), this new form of prion disease appeared to be linked to the consumption of BSE contaminated meat products. The two main differences between sporadic and vCJD are the dramatic decrease in age at onset of symptoms (29 years) and the increased duration of symptoms (16 months) prior to death [61]. Some additional symptoms that are typically rare in sporadic CJD like involuntary movements and sensory symptoms have been observed in 100% and 50% of symptomatic vCJD patients, respectively [62].

 As of 2003, the number of autopsy-confirmed cases of vCJD was 153, and currently the overall number of individuals infected is estimated at 200 (http://www.bseinfo.org/). While these numbers are low, the number of people actually infected with vCJD and not showing symptoms is likely much higher. Due to incubation times that can last decades, there is concern that there may be a population of asymptomatic carriers harbouring infectious PrP in the absence of observable symptoms. Without an accepted premortem diagnostic tests, health professionals are unable to identify these individuals. This creates a potentially dangerous situation as evidence suggests that vCJD can be transmitted via contaminated blood products [63], infectious cadaveric tissues, and contaminated surgical instruments [64].

## 8. Understanding Prion Diseases

Subsequent to the observation that TSE neurodegeneration reflects an infection, an exhaustive search for the traditional signs of infectious agent began. Populations of T and B cells were analyzed in mouse models of scrapie infection, but no differences between the experimental and control groups were detected [65]. Experiments looking at the potential influence B cell maturation on scrapie infections showed no difference in disease incubation or progression between wild-type mice and mice deficient in mature B cells [66]. Attempts to detect a scrapie-induced humoral response also failed [67]. It was determined, however, that scrapie infections involved the lymphoreticular system, independent of the site of inoculation, as high scrapie titers were detectable in spleen and other lymphoid organs [68, 69]. Thus, while many researchers had shown scrapie to be transmissible disease, initial efforts to characterize it from an immunological perspective indicated scrapie behaved in a fashion distinct from traditional infectious agents.

With scrapie infection studies producing perplexing results, Stanley Prusiner focused on characterizing the infectious component. Sedimentation experiments demonstrated that the scrapie agent aggregated with cellular components [70] and was stable in nonionic and anionic detergents [71]. Other experiments revealed it to be a hydrophobic molecule, a characteristic that complicates purification procedures and allows for extreme heat stability [72]. Several key pieces of evidence indicated that one of the required components of infectivity was a protein; digestion with proteinase-K or protein denaturation via sodium dodecyl sulfate (SDS), trypsin, phenol, urea, and chaotropic salts all led to a loss of infectivity [73].

Early attempts to identify the genetic component associated with the scrapie agent began with pH stability studies. Infectious material was inactivated by alkali pH, whereas acidic treatments had no effect [71]. Because alkali conditions disrupt both proteins and nucleic acid, the results were unable to rule out the presence of nucleic acids in association with the scrapie agent [71]. Further experiments, in particular ones that determined the scrapie agent was resistant to nuclease digestion and ultraviolet irradiation, suggested there was no nucleic acid content in the scrapie agent [71]. Collectively, this extensive molecular analysis determined the unique nature of the scrapie agent. Specifically, its resistance to nucleic acid degradation treatments, small size, and resistance to heat inactivation suggested the scrapie agent was a new type of infectious agent. Prusiner labelled these agents as “prions” which he defined as “small proteinaceous infectious particles which are resistant to inactivation by most procedures that modify nucleic acids” [71].

## 9. Cellular PrP

The human version of PrP^C^ is a 253 amino acid (aa) protein which is encoded by the prion gene *Prnp* located on the short arm of chromosome 20. In addition to regulatory regions such as heat shock elements, *Prnp* is composed of three exons [74]. The discovery that the entire open reading frame is contained within a single exon (the third one) helped eliminate the possibility that the infectious form of the protein results from alternative RNA splicing [75]. PrP^C^ mRNA is constitutively expressed in many different tissues with the highest levels of expression in neurons [76], but there are also significant levels of expression in the heart [77], skeletal muscle [78, 79], lymphoid tissue/white blood cells [80, 81], gut tissues [82], and reproductive tissues such as the testes and uterus [83]. Little is known regarding the regulation of PrP^C^ expression other than the entire first intron is required for full promoter activity [84], and heat shock elements are responsible for upregulating expression during periods of cellular stress [85].

## 10. PrP^C^ Structure

The cellular prion protein (PrP^C^) is a highly conserved glycosyl-phosphatidyl-inositol (GPI-) anchored membrane protein. Before PrP^C^ is delivered to the outer cell membrane, and it is targeted to the endoplasmic reticulum (ER) where the N-terminal signal peptide is cleaved and a GPI anchor is added to the C-terminus at serine 231, creating a 209 amino acid protein [86]. At the outer membrane, PrP associates with lipid rafts and is cycled between the cell surface and the endocytic compartment at approximately 60-minute intervals with 95% of the internalized protein being recycled back to the cell surface [87]. Nuclear magnetic resonance (NMR) studies have determined that PrP^C^ has a flexible N-terminal unstructured domain which contains four octapeptide-repeat regions and a globular C-terminal domain consisting of three *α*-helices and two antiparallel *β*-sheet structures [88]. There is a hydrophobic sequence in the middle of the protein from aa 113–135 which some suggest may serve as a transmembrane domain in some prion isoforms [89]. Additionally, there is a single disulphide bond between cysteine residues 179 and 214 and two N-glycosylation sites at asparagine residues 182 and 198 [31].

 Prion structural comparisons among species indicate great similarity, which is anticipated with 90% sequence homology [90]. Cervid PrP, however, contains a unique and well-defined rigid loop located between *α*-helix 2 and the connecting *β*-strand (aa 166–175). This same region in other species is flexible and disordered. Further investigations revealed that the rigidity of the loop in cervids is due to a two-amino acid substitution S170N and N174T [7]. Structural differences in PrP^C^ have been shown to influence a species susceptibility to PrP^Sc^. Consequently, it has been hypothesized that the rigid loop structure in cervid PrP may be a contributing factor to the rapid horizontal CWD transmission [7].

## 11. PrP^C^ Function

Despite the highly conserved sequence across species and ubiquitous expression within the body, little is known about the function of PrP^C^. It has been hypothesized that PrP^C^ may be involved in multiple functions: copper metabolism, due to its binding affinity for copper; neuroprotective function due to its antiapoptotic activity [91, 92]; signal transduction [93, 94]; synapse formation/function [95]; neuritogenesis [96].

 Copper has an important physiological role in all organisms, functioning as a cofactor in processes such oxidative stress protection, blood clotting, normal cell growth and development, respiration, and iron transport [97]. Copper deficiencies in humans have been linked to serious diseases which include Menkes syndrome [98], and various neurodegenerative disorders such as Alzheimer's disease (AD), amyotrophic lateral sclerosis (ALS), and transmissible spongiform encephalopathies (TSEs) [99]. Numerous studies have shown that the histidine-containing octapeptide repeat region of the PrP^C^ protein is capable of binding up to four Cu^2+^ ions [100]. These copper ions stimulate a conformational change in PrP^C^ resulting in a structure that has some characteristics which are similar to the infectious conformation, including a higher *β*-sheet content, increased protease resistance, and propensity to aggregate, but the structure is distinct from the PrP^Sc^ isoform [101]. It was also found that micromolar concentrations of copper induce endocytosis of cell-surface-associated PrP^C^ [102]. Consequently, it has been suggested that PrP^C^ has a role in copper uptake/efflux and may also serve as a copper reservoir at the cell surface without stimulating endocytosis [103].

One model which has been used to explain the pathogenesis of several neurodegenerative disorders is chronic oxidative stress. Impairment of mitochondrial function, increased oxidative damage, defects in the ubiquitin-proteosome system, protein aggregation, altered iron metabolism, excitotoxicity, and inflammation have all been linked to oxidative stress in the brain [104]. Studies of oxidative stress in neurons suggest that PrP^C^ protects against reactive oxygen species (ROS) [105]. Brown et al. proposed three possible mechanisms by which this may occur. One explanation is that the PrP^C^ acts directly on the ROS via a copper-dependent superoxide dismutase (SOD) activity [106]. SODs are enzymes which remove ROS by converting them into a more stable H_2_O_2_ [104]. A second theory is that PrP^C^ acts indirectly by upregulating activities of molecules like Cu-Zn SOD [103]. The last hypothesis posits that PrP^C^ may prevent activation of ROS-induced apoptosis [104]. 

*In vitro* signalling analysis and prion knockout (PrP^0/0^) studies indicate that PrP^C^ mediates interaction between several signalling transduction pathways including protein kinase A, Fyn, phosphatidylinositol 3-kinase (PI3K)/Akt, and mitogen-activated protein kinase/extracellular signal-regulated kinase (MAPK/ERK). These pathways are known to induce neuronal survival [94, 107]. Studying induced ischemic injuries in neuronal tissue of PrP^0/0^ and wild type (WT) mice, it was demonstrated that the absence of PrP^C^ exacerbates ischemic brain injury. The authors hypothesized this was due to reduced activation of the antiapoptotic pathway PI3K/Akt which in turn resulted in increased caspase-3 activity [108].

 Protein localization studies determined that PrP^C^ was present in the presynaptic region of the axon terminus [109]. This observation is supported by evidence of synapse loss, PrP^Sc^ accumulation at the synaptic terminal [110], and abnormal electrophysiological recordings during prion disease progression [111]. In the terminal stages of disease, accumulation of PrP^Sc^ in synaptosomes was found to coincide with alterations in the gamma-aminobutyric acid (GABA) system which is involved in inhibition of excitatory glutamatergic transmission [112]. The synaptic involvement of PrP^C^ may also be linked to circadian rhythms [113] and the impaired hippocampal-dependent spatial learning phenotype in PrP-deficient mice [114].

## 12. Infectious PrP^Sc^

The infectious component of prion diseases appears to be an insoluble *β*-sheet-rich version of the normal, nonpathogenic *α*-helical PrP^C^. While the primary structures of the two conformations are identical, differences in secondary and tertiary structure account for the unique properties of PrP^Sc^ [115]. Unlike PrP^C^, the infectious isoform is resistant to protease K (PK) digestion and readily forms insoluble aggregates [116]. These aggregates are composed of hydrolyzed fragments of the PrP^Sc^ protein, referred to as PrP 27–30 to reflect their molecular weights that range between 27 to 30 kDa. While X-ray and NMR structures have been determined for PrP^C^, the tendency for PrP^Sc^ to aggregate renders the isomer unsuitable for most types of structural analysis. Through techniques such as circular dichroism, which offer less specific structural information, it has been determined that there is an increase in *β*-sheet content from 3% to 42% as the conformation changes from PrP^C^ to PrP^Sc^ [117].

Although PrP^Sc^ is generally described as the infectious conformation, subtle variations exist which make identifying the exact infectious component more challenging. One variation, referred to as PrPSensitive (PrP^Sen^), is more susceptible to PK digestion than another variation, PrPResistant (PrP^Res^). While most PrP^Sc^-infected tissues contain PrP^Res^, its presence is not an absolute requirement for infectivity [118, 119]. PrP^Sen^ has also been identified in scrapie-infected tissue, making it difficult to discern which version is responsible for infectivity [120, 121]. There is evidence for the existence of multiple PrP^Sc^ isoforms, also referred to as strains, which possess unique infectivity, pathology, neurotropism, and biophysical traits.

## 13. Prion Strains

Strains within traditional pathogens, such as bacteria and viruses, are determined by nucleic acid sequences in their genome. The term “strain,” when used in the context of prion disease, refers to phenotypic differences such as variations in the pattern of aggregate deposition, incubation times, neuronal tissue tropism, and pathological morphology [122]. The mechanism of strain formation in prions is not attributable to differences in the encoding of primary PrP structure. Rather, these differences are thought to result from the conformational flexibility of the PrP^Sc^ structure which leads to exposure of distinct cleavage sites, differing stability in the presence of denaturing compounds [123], and altered ratios of di-, mono-, and unglycosylated forms [124]. The strain of an incoming infecting PrP^Sc^ molecule appears to be imprinted in all of the subsequent PrP^Sc^ proteins produced during the conversion of the host's cellular PrP [125]. It has been demonstrated that different strains can be passaged serially through inbred mice with identical PrP gene sequences [126]. Strains have also been reisolated in mice following passage through other species with differing PrP primary structures [127].

 It has been shown that prion strain characteristics contribute to species barriers. Species barrier is a concept whereby the transmission of prion diseases between different species is much less efficient than within species [126]. For example, when species B is infected with prion from species A, the infectivity rate is low and disease progression, in successfully infected animals, is slow and unpredictable. In contrast, when species A is infected with prions from species A, infection rates are high and progression is more rapid with a more consistent disease course among animals. Interestingly, following the passage of prion from species A to B, subsequent passages of the same prion to another B animal now have the same disease progression as if the prion had originated from a species B animal. The type of strain influences how easily this species barrier is broken. For example, the prion strain responsible for the BSE crisis was transmissible to a variety of species, including humans [127].

## 14. Transmission of Infectious PrP

Natural transmission of TSEs likely occurs though ingestion of contaminated material (skin, dirt, decomposed carcasses, urine, contaminated placentas, and faeces) or by direct contact between animals [128]. Transmission of prion diseases between different species has been shown to be much less efficient than within the same species. This suggests that TSEs are limited by a species barrier further trans-species experiments have demonstrated that transmission does occur but that incubation times may be greatly extended so that clinical disease may or may not occur during the natural lifespan of the affected animal [129]. The concept of prion strains complicates the understanding of TSE transmission in that certain strains of PrP^Sc^ derived and transmitted within the same species can result in either the development of an asymptomatic carrier or the onset of clinical disease [130].

## 15. Pathogenesis of Prion Diseases

The formation of PrP^Sc^ is thought to occur through an interaction whereby an infectious PrP^Sc^ particle induces refolding/misfolding of PrP^C^ to PrP^Sc^. PrP^C^ is an absolute requirement for infection and disease. Challenge of PrP-deficient mice with PrP^Sc^ material produces no accumulation of infectious material or disease. When PrP^C^ expression is restored, typical prion disease pathology is observed [131]. Transgenic mice engineered such that PrP was produced without the GPI anchor failed to develop clinical symptoms following scrapie inoculation. Unlike PrP knock-out mice, however, the GPI anchor transgenic mice still produced PrP^Sc^ and amyloid plaque aggregation [132]. This illustrates the need for membrane-anchored PrP^C^ for pathology to develop. 

One research group investigated whether PrP^C^ expression was necessary on all types of brain cells (neurons, astrocytes, and oligodendrocytes) for formation of PrP^Sc^, development of disease, and transmission of infectivity. Several transgenic mouse lines were created with PrP^C^ expression restricted to specific cell lineages. Mice with only neuronal PrP expression supported prion infection and developed clinical disease [133]. In contrast, when PrP^C^ expression was restricted to oligodendrocytes, then prion disease failed to develop [134]. Mice with PrP expression restricted to astrocytes also developed disease symptoms despite the lack of neuronal PrP^C^ [135]. It was suggested that deposition of PrP^Sc^ in close proximity to neurons and their processes was sufficient to induce pathology [136]. This would seem to contradict the work with soluble PrP^C^ which suggested that membrane-anchored PrP was necessary for neuronal pathology [132]. Alternatively, PrP^Sc^ may have indirect effects on neurons by altering the function of associated cells. 

Because the definitive function of PrP^C^ has yet to be determined, the pathological mechanisms of PrP^Sc^ are also uncertain. One argument offers that PrP^Sc^ causes a “gain of function” whereby the presence of the misfolded protein adds a neurotoxic function. The opposing hypothesis is that PrP^Sc^ causes a “loss of function.” Some research has also shown that PrP^Sc^ itself is not neurotoxic due to the lack of correlation between PrP^Sc^ deposition and disease severity. It has been suggested that the conversion of PrP^C^ to PrP^Sc^ is the key component in pathogenesis rather than the accumulation of PrP^Sc^ aggregates [137].

 There are two main theories about the mechanism by which PrP^Sc^ induces the misfolding of PrP^C^, the template-directed refolding model, and the nucleated polymerization model. The first model postulates that incoming PrP^Sc^ initiates a catalytic cascade using PrP^C^, or a PrP intermediate, as a substrate for conformational conversion into a new *β*-sheet-rich protein. This new PrP^Sc^ protein then serves to convert the next PrP^C^ molecule as the cycle continues. The refolding mechanism is kinetically controlled by a high activation energy barrier which prevents spontaneous conversion at detectable rates. It is thought that this energy barrier is lowered by the formation of an intermediate PrP^C^-PrP^Sc^ heterodimer complex which facilitates the full conversion of PrP^C^, with the help of a chaperone molecule, into the new PrP^Sc^ conformation [124].

 The second model is based on a thermodynamically controlled nucleated polymerization reaction. This is a noncatalytic event whereby conversion of PrP^C^ to PrP^Sc^ is technically a reversible process. At equilibrium, however, the natural cellular PrP isoform is highly favoured. The isoform conversion only occurs when a native PrP protein monomer comes into contact with an already formed PrP^Sc^ crystal seed or aggregate. Successful refolding into the PrP^Sc^ isoform is stabilized once the new protein is added onto the preformed seed. This model implies that infectivity requires the presence of PrP^Sc^ in oligomer form and that monomers are not infectious [124].

## 16. Peripheral Amplification

TSE infections are usually established following oral ingestion of infectious PrP^Sc^ material. Once in the digestive tract, it is proposed that there are three potential routes for PrP^Sc^ to cross the mucosal barrier. The first utilizes a specific subset of intestinal epithelial cells called microfold cells or M cells. These cells possess a thin apical membrane made up of mircofolds and an invaginated basal membrane which houses various immune cells. The thin membrane allows the transport of many types of bacteria, viruses, and other proteins, including prions, across the epithelial layer where they then encounter lymphocytes and dendritic cells [138]. This interaction between the immune cells and pathogenic antigen usually serves to stimulate a mucosal immune response. While the M cells facilitate uptake and transfer of Ag to the immune system, they may also serve as a portal of entry for pathogens like prions which are not degraded and do not stimulate an immune response. Therefore, prions are left to continue on their route of infection and interact with other cells, alternatively, while in the intestinal lumen, PrP^Sc^ can be degraded by digestive enzymes into smaller protease-resistant fragments. These fragments may form complexes with ferritin which are transported across the intestinal epithelium by a ferritin-dependent endocytosis mechanism [139]. A third mechanism of uptake has been proposed based on observations made during bacterial research and has yet to be directly linked to TSE transport. This involves direct sampling of prion protein by migratory bone marrow-derived dendritic cells which can travel from the blood vessels to the inner surface of the intestinal wall. These DCs then sample luminal contents, including prions, and trapped protein is transported to the lymphoreticular system which includes the draining mesenteric lymph nodes [140].

Immunohistochemistry reveals that once across the mucosal barrier, PrP^Sc^ accumulation occurs in a variety of lymphoid tissues including palatine tonsils, spleen, lymph nodes, and Peyer's patches [141]. More specifically, PrP^Sc^ replication and accumulation occurs within tingible body macrophages located in B-cell follicles and later on the plasmalemma of the follicular dendritic cells (FDCs) [16]. This amplification phase, particularly within the Peyer's patches, is important for PrP^Sc^ transfer to the nervous system.

Kitamoto et al. were instrumental in establishing the relationship between B cells, FDCs, and prion disease progression. B-cell-deficient mice were successfully infected with prion material via intracranial (IC) challenge, but failed to show any signs of disease following intraperitoneal (i.p.) challenge. Further investigation of the i.p.-inoculated mice revealed that PrP^Sc^ failed to accumulate in the spleen and brain despite having normal levels of FDCs [142]. Based on previous studies which illustrated that FDC development was a B-cell-dependent process, the Kitamoto group concluded that removal of the B-cell population had prevented maturation of the FDC population. It became evident that FDCs are essential for prion replication and disease progression. This was verified by reconstituting B cells in B-cell deficient mice, which had been given prion material i.p., and restoring susceptibility to prion disease.

Following the linkage of FDCs and prion replication, studies discovered a link between the inflammatory process (involving FDCs, B cells, macrophages, and other immune cells associated with germinal centers) and accumulation of PrP^Sc^. In the presence of inflammation, the upregulation of FDC cytokines such as lymphotoxin resulted in an ectopic induction of PrP^C^-expressing FDCs [60]. As a result, nonlymphoid organs undergoing inflammation, for example, kidney, liver, and pancreas, show signs of prion accumulation. In contrast, these organs are prion-free under normal conditions [143]. Experiments have shown that in symptomatic and asymptomatic scrapie-infected mice, with nephritis, prion infectivity was also detectable in the urine [144]. Similar experiments in sheep with mastitis showed that infectivity was present in colostrum and milk [60]. This concept is problematic in the context of wild/farmed animal populations where PrP^Sc^-infected individuals suffering from inflammatory processes in excretory organs may shed PrP^Sc^ material into the environment and increase disease transmission.

## 17. Neuroinvasion

The mechanism by which PrP^Sc^ travels from lymphoid tissue to the nervous system is not well understood. Experimental conditions that either remove or increase lymphoid innervation show that increased lymphoid innervation correlates with faster neuroinvasion [145]. The proximity of PrP^Sc^ expressing FDCs to the sympathetic nerve endings has been shown to influence rates of neuroinvasion. Whether there is passive diffusion of infectious PrP molecules or a facilitated transport system between FDCs and nerve endings is unknown [58].

The enteric nervous system (ENS) has been identified as the first neural tissue to be invaded by PrP^Sc^ following the peripheral amplification phase [141]. The ENS, a member of the autonomic nervous system, innervates the gut and is made up of two main networks, Meissner's plexus, located in the gut submucosa, and Auerbach's plexus, which runs between the circular and longitudinal muscle layer and extends from the esophagus to the rectum [146]. As a component of the autonomic nervous system, the ENS is directed by the sympathetic and parasympathetic input from the central nervous system via efferent nerves which connect to the enteric plexi [146]. Following infection of the ENS, PrP^Sc^ travels to the brain using mainly the sympathetic pathway via the Vagus nerve to the dorsal motor nucleus in the medulla oblongata, or the parasympathetic pathway via the splanchnicus nerve to the intermediolateral column of the spinal cord [147].

## 18. Neurodegeneration

The mechanisms for prion-induced neurodegeneration are closely related to the proposed functions of PrP^C^. This is to be expected as the TSE disease process is essentially the conversion of PrP^C^ to PrP^Sc^ which leads to a reduction in the number of functioning cellular prion proteins, thus sparking the debate about whether prion pathology is due to a gain of a neurotoxic function or the loss of a neuroprotective function. Hur et al. proposed five mechanisms of prion neurodegeneration: increased oxidative stress and mitochondrial dysfunction; disruption of iron metabolism, altered calcium metabolism; increased inflammatory activity of cytokines, chemokines, and nuclear factor-kappa; finally, apoptosis [148].

 It has been observed in TSE-infected animals that there is reduced activity of manganese SOD. This mitochondrial enzyme is responsible for converting superoxide anions into less reactive species. The resulting accumulation of ROS leads to ischemic cell injury and subsequently apoptosis or necrosis. Additional mitochondrial deficits, such as decreased cytochrome-c oxidase and ATPase activity, increased lipid peroxidation, and structural abnormalities, have been detected in hippocampal and cerebral cortical neurons of TSE-infected hamsters [149].

 Increased iron metabolism within the brain (favouring a redox state of Fe^+3^), as a result of a TSE infection, is involved in exacerbating ROS injury by converting harmful free radicals, such as a superoxide anion, into an even more highly reactive hydroxyl radical [148].

 Proper calcium regulation is essential for the normal functioning of the CNS. Variations in intracellular calcium concentrations facilitate the coordination of electrochemical signals, neurite growth, synaptogenesis, synaptic transmission, cell survival, and plasticity [150]. *In vitro* experiments showed that in PrP^Sc^-infected cells, there was a downregulation of N-type voltage-gated Ca^2+^ channels resulting in reduced cytosolic Ca^2+^ [151]. PrP^Sc^ aggregates that accumulate in the synaptic cleft may physically interrupt synaptic transmission of electric potentials created by Ca^2+^-activated potassium channels, or compromise the stability of newly formed synapses [150]. This can result in excitotoxicity [152] and ischemic brain damage [153]. The interference with the development of long-term potentiation also has negative effects on processes such as learning and memory [150].

## 19. Prion Diagnostics

With the lack of accepted premortem diagnostic tests, health professionals have to rely on clinical examinations for diagnosis of TSEs [154]. Definitive confirmation, however, can only be made by postmortem histological or biochemical analysis of brain tissue [155]. While histological assays are accurate, they are time consuming, labour intensive, and low throughput. Development of new biochemical tests has allowed multiple samples to be processed in a few hours. The majority of the tests have detection limits ranging from 0.5 pmol to 20 pmol and utilize immunodetection of the PrP^Sc^ isoform or proteolysis to distinguish PrP^C^ from the infectious conformation [154].

 To enable the detection of peripheral PrP^Sc^ in preclinical cases, Saborio et al. developed a method of PrP^Sc^ amplification called protein misfolding cyclic amplification (PMCA) [156]. Analogous to the polymerase chain reaction method used to amplify DNA, PrP^C^ of the same species is added to a sample suspected to contain small quantities of PrP^Sc^ and incubated to allow for the expansion of PrP^Sc^ aggregates. These new aggregates are broken up using sonication to form more PrP^Sc^ seed molecules and are reincubated in the presence of added PrP^C^. After several amplification cycles, the amount of PrP^Sc^ is several millionfold higher than in the original sample [157], allowing detection using standard molecular techniques like Western blots. Concerns have been raised regarding the specificity of this technique after it was shown that PrP^Sc^ can be generated from samples lacking PrP^Sc^ [158]. A lack of specificity would limit the usefulness of PMCA as a diagnostic test.

 The discovery of PrP^Sc^ infectivity in blood from vCJD patients [158, 159] emphasized the need for a blood test for prions. The first detection system was developed in 1996 and consisted of capillary electrophoresis and a competitive immunoassay which detected a PK-resistant C-terminal sequence of PrP [160–162]. Despite several revisions, the method was not suitable for routine testing [163]. More recent initiatives involve the use of a 15B3 prion-specific antibody developed by Prionics [164, 165] and a Seprion resin manufactured by Microsense. Both systems concentrate PrP^Sc^ so it is detectable by ELISA and flow cytometry. Another group designed fluorescent labelled palindromic peptides to discriminate PrP^C^ from PrP^Sc^. When the labelled peptide binds to PrP^Sc^, a shift in conformation modifies the fluorescent properties of the label [166]. Despite the advances in blood diagnostics, the strict guidelines governing diagnostic requirements prevent their acceptance for use in an official capacity.

## 20. Historical Approach to Prion Therapy

For the last fifty years, there have been ongoing efforts to identify effective prion therapeutic agents. Typically, these agents have targeted either PrP^C^, PrP^Sc^, or the conversion process. These efforts were energized with the emergence of vCJD in the 1990s and the discovery that prion infectivity can be transmitted via blood, surgical instruments, and transplant tissues from asymptomatic carriers [167]. Investigations of potential therapies have been primarily conducted in three experimental systems: cell-free *in vitro* conversion assays, cell-based models, or animal models [137]. The cell-free method involves the addition of a PrP^Sc^ seed molecule to PrP^C^ substrate in the presence of a potential therapeutic agent. Therapeutic potential is evaluated based on the ability of the molecule to interfere with the conversion process. Cell based research utilizes cell lines such as N2a (mouse neuroblastoma cells) to assess the ability of a given therapeutic agent to either prevent infection of the cells by prions, or to clear PrP^Sc^ from a chronically infected cell line like ScN2a. These cell models allow for the analysis of some very basic aspects of prion pathogenesis such as isoform conversion, cytotoxicity, apoptosis, and abnormal cell signalling. Animal models, which make up the third experiment system, tend to be the most expensive, yet informative, model as many characteristics of a potential therapeutic can be analyzed. These include its ability to influence incubation phase of infection, severity of symptoms, pathology, immune response, and survival time and rate. Animal models are complicated, however, by issues such as genetic variability between and within species, inconsistent models of infection, and lengthy incubation periods.

 Dating back to the 1960s, the main area of antiprion research was focused on chemotherapeutic agents [137]. As it was initially thought that prion diseases were the result of a slow virus, this biased the nature of the therapeutic agents which were investigated. Typical TSE targets/goals for chemotherapeutic agents have been sterilization of sources of infection; prion prophylaxis; interruption of PrP^C^ conversion during peripheral amplification; prevention of neuroinvasion; reduction of PrP^Sc^ accumulation [168]. Countless agents have been tested including polysulphated polyanionic compounds, glycosaminoglycans, sulphonated dyes, quinacrines, metal chelators, tetrapyrroles, polylene antibiotics, tetracyclic compounds, and *β*-sheet breaker peptides [137, 168]. Very few therapies have shown significant success *in vivo*, particularly if delivered after symptoms had already developed [169]. This likely reflects the inability of many of these molecules to cross the blood-brain barrier which is required to interfere with prion-related pathogenesis in the CNS. However, even compounds which are able to penetrate the CNS, like curmunin, quinacrine, quinolones and polyene antibiotics, failed to improve symptoms of late-stage TSE disease [168].

 The appreciation that prion diseases result from the misfolding of a self-protein, rather than a slow virus, impacted the efforts to develop therapeutic agents. Conceptually with a traditional infectious disease, there is a clear distinction between the host and pathogen. In contrast, the prion diseases, which result from the subversion of a normal and omnipresent component of the body, are conceptually closer to a cancer than a traditional infectious. Similar to challenges faced in cancer therapeutics, the issue of specificity takes on elevated importance as the distinction between host and pathogen becomes less clear.

## 21. Immunotherapy of Prion Diseases

**Figure 1:**
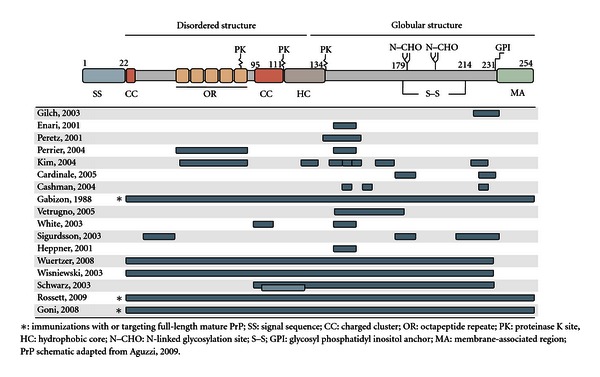
Regions of PrP targeted in the various immunotherapeutic investigations.

**Figure 2:**
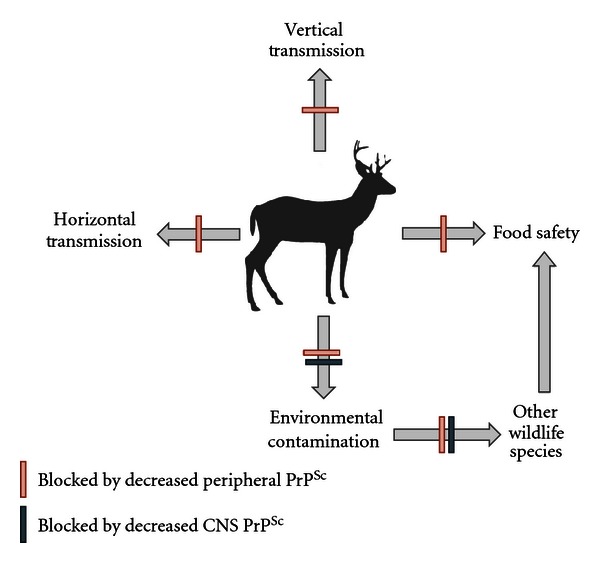
Potential points of impact of a prion vaccine. An effective CWD vaccine should decrease peripheral and CNS loads of individual animals, leading to decreased transmission both within and outside cervid populations, and decreased environmental shedding. These factors would lead to an overall reduction in available infectious prion and benefit both wild and food animals, as well as humans.

While prion diseases represent a novel paradigm of infectious disease, there nevertheless appears to be an opportunity to apply traditional medicinal approaches to impact disease susceptibility and progression. In particular, there is proof-of-principle evidence from a number of models that a vaccine may be an effective tool to control prion diseases. There are numerous attempts to develop a prion vaccine which have focused on different epitopes, strategies of formulation/delivery which have been tested in a number of models of disease (Figure 1). For clarity, these efforts are grouped here according to investigations which evaluate the therapeutic potential of particular antibodies with *in vitro* systems and *in vivo* systems, which include subcategories of passive and active immunizations. The critical findings of these studies are presented in (Tables 2 and 3) for *in vitro* and *in vivo* investigations, respectively. An illustration of the various regions of the PrP protein which have been targeted in the various investigations is presented in (Figure 2).

## 22. *In Vitro * Immune Therapy

Conversion of PrP^C^ to PrP^Sc^ occurs at, or near, the cell surface [170, 171]. This provides an opportunity for antibodies to bind and prevent the interaction and conversion of PrP^C^ by PrP^Sc^. Such a strategy might be used as immunoprophylaxis to prevent infection of animals exposed to PrP^Sc^ or as therapy to treat infected animals, serving to delay or stop the progression of disease and minimize the shedding of infectious PrP^Sc^. This approach is challenged by the fact that PrP^C^ is a ubiquitously expressed, endogenous protein making it very difficult to stimulate an immune response. This is the result of an immunological phenomenon known as tolerance which occurs when the immune system recognizes a protein as being “self.” Consequently, immune cells, such as T and B cells, which have receptors specific to that particular protein, are deleted or prevented from initiating an immune response towards that particular protein [172]. Overcoming tolerance to PrP^C^ remains one of the biggest challenges in prion vaccine research [173]. 

Some of the first demonstrations that antibodies could be used to neutralize prions came from experiments that demonstrated that *ex vivo* incubation of a prion inoculum with anti-PrP^C^ polyclonal antibody, prior to inoculation, resulted in a 2-log reduction in infectivity [174]. Further work was done to identify the region of PrP involved in the interaction with PrP^Sc^ resulting in isoform conversion. This was done through the application of mAb 3F4 (specific for aa 109–113) [175] as well as polyclonal sera to peptides (corresponding to aa 23–33, 90–104, 143–156, and 219–232) in efforts to prevent PrP^Res^ formation in a cell-free conversion system [174]. Antibodies to the region 219–232 were found to disrupt the formation of new PrP^Res^. The same effect was not observed during incubation with PrP^Res^ seed. Furthermore, when PrP^Sen^ Abs and peptide 219–232 were coincubated, the Ab bound to the peptide enabling the uninterrupted formation of PrP^Res^. This illustrated that the inhibition of PrP^Res^ formation was due to the direct binding of the Ab to the 219–232 region [176].

 Preliminary immunotherapy research in 2001 showed that prolonged exposure (6 weeks) of ScN2a cells to mAb 6H4 (specific for aa 144–152 on the prion protein) resulted in the complete clearance of PrP^Sc^ offering evidence that anti-PrP Abs have the potential to interfere with the prion disease process [177].

 That same year, Peretz et al. used recombinant antibody antigen-binding fragments (Fabs) to prevent PrP^C^ conversion in ScN2a cells infected with PrP^Sc^. They observed a direct relationship between the amount of antibody binding to surface PrP^C^ and the inhibition of PrP^Sc^ formation. Their most effective Fab, D18, not only stopped PrP^Sc^ formation but also cleared preexisting PrP^Sc^ infection. In related challenge experiments, mice infected with scrapie-infected cells preincubated with the antibodies survived more than three months longer than those receiving untreated cells. The success of D18 was attributed to its ability to bind cell surface PrP^C^ molecules [179].

In 2004, two antibodies, SAF34 (specific for the octarepeat region of PrP^C^) and SAF61 (specific for 144–152 of both PrP^C^ and PrP^Sc^), were studied in neuroblastoma cells overexpressing PrP^C^ with and without PrP^Sc^ infection [180]. When used independently, each antibody decreased levels of PrP^C^ and PrP^Sc^ in uninfected and infected cells, respectively and when used together had a synergistic effect. The authors suggested that SAF61s acted by promoting clearance of PrP^C^ to remove the substrate required for PrP^Sc^ production. SAF34, although showing a similar end result, functioned through a different mechanism by blocking interaction between PrP^C^ and PrP^Sc^ [177, 179]. The epitopes recognized by SAF34 and SAF61 correspond to a region of PrP thought to be involved in an interaction with the laminin receptor [192]. Consequently, the binding of SAF antibodies to PrP may also interfere with this process [180].

 Using PrP^0/0^ mice, Kim et al. produced a panel of antibodies to either recombinant PrP or PrP^Sc^ [181] and evaluated the ability of these antibodies to protect ScN2a cells from infection. They concluded that antibody-mediated protection was not in the specificity of the epitope, but rather in the capacity of antibody to bind cell surface PrP^C^ [193]. At the end of its natural cycle, surface-expressed PrP^C^ becomes internalized via clathrin vesicles and then enters the degradation pathway [194, 195]. It is during this internalization process, at the level of the cell membrane, that the conversion from PrP^C^ to PrP^Sc^ is believed to occur [171, 196]. The authors hypothesized that the binding of mAb to surface PrP^C^ prevents internalization to inhibit the conversion process [193].

 In contrast to targeting surface PrP, Cardinale et al. developed antiprion single-chain antibody fragments (scFv) with an endoplasmic reticulum retention signal to target intracellular PrP. Stable expression of these scFvs in nerve growth factor differentiating PC12 cells inhibited PrP^C^ translocation from the ER to the cell surface and prevented PrP^Sc^ accumulation [182]. They later applied this concept to an *in vivo* model. Lysate from wild-type and scFv (8H4)-expressing PC12 cells was injected intracerebrally into C57BL mice, 35 days after exposure to scrapie. Mice that received wild-type PC12 cells succumbed to prion disease, whereas only 2/10 mice in the scFv (8H4) group were infected. At 300 days after infection, the eight mice of the experimental group remained symptom-free with no detectable histopathology [183].

## 23. *In Vivo * Immunization

The necessity for extracerebral PrP^Sc^ to undergo amplification in the periphery prior to neuroinvasion provides another opportunity for prophylaxis. However, due to immunological tolerance to PrP^C^, attempts to stimulate humoral responses against prion proteins *in vivo* have proven challenging. In 1993, Prusiner's group demonstrated that it is possible to raise antibodies to PrP in *Prnp* ^0/0^ mice. Aguzzi et al. speculated that the use of genes encoding high-affinity anti-PrP antibodies produced in *Prnp* ^0/0^ mice might be able to redirect B-cell responses in mice expressing PrP^C^. They took *Prnp* ^0/0^ and *Prnp* ^+/0^ populations of mice and transgenically introduced genetic sequences which encoded for the heavy and light chains of 6H4, an mAb specific for PrP^C^. By four weeks of age, the *Prnp* ^0/0^ mice consistently produced high anti-PrP titers. In contrast, mice expressing PrP (*Prnp* ^+/0^) developed anti-PrP antibodies more slowly. Unresponsiveness of B cells likely corresponds to the level of self-antigen presented [197] and B-cell receptor avidity/affinity [198]. Therefore, the delay in production of anti-PrP in *Prnp* ^+/0^ mice may be due to negative selection of B-cells expressing 6H4 epitopes with high avidity for PrP^C^ [186]. Consequently, the 6H4 produced by *Prnp* ^+/0^ mice may have different binding properties than 6H4 produced by the *Prnp* ^0/0^ mice. All mice were inoculated i.p. with RML scrapie. Spleens from the nontransgenic *Prnp* ^+/0^ group showed PrP^Sc^ accumulation, while the *Prnp* ^+/0^-6H4 group did not. Brain samples, used to assess the ability of PrP^Sc^ to spread to the CNS, showed identical results to that of the spleen. To rule out unintended mechanisms of PrP^Sc^ protection, Western blots were used to determine whether the presence of 6H4 was causing reduced expression of PrP^C^. Equal levels of PrP^C^ protein in both the transgenic and nontransgenic *Prnp* ^+/0^ mice indicated this was not the case. Instead, it appears that protection was occurring via masking of PrP^C^ sites critical for interaction with PrP^Sc^. Interestingly, as PrP^C^ is an abundant self-protein, the risk of inducing autoimmunity is always a concern; however, no obvious signs of autoimmune disease were detected in this study [186]. 

While the presence of anti-PrP antibodies in the peripheral compartments does not appear to have any negative consequences, Solforosi et al. investigated the effect of anti-PrP^C^ antibodies on neuronal cells *in vivo*. PrP^C^ D13 mAbs were injected into the hippocampus of C57BL/10 mice. Within 24 hrs, antibody-induced cross-linking of cell-surface PrP^C^ resulted in extensive apoptosis of neurons in the hippocampal and cerebellar regions [199]. That administration of monovalent Fab fragments from D13, via the same methods, produced apoptosis at a much reduced rate suggests that the cross-linking event was most likely the cause of apoptosis. In contrast to the previous results, experiments with a D18 mAb specific for a region involved in PrP^C^-PrP^Sc^ interaction did not trigger apoptotic cell death. This may be because D18 was ineffective at cross-linking PrP^C^, or it obscured the region of PrP^C^ which binds to a cofactor molecule necessary for apoptosis signalling [199].

## 24. Passive Immunization

Further enthusiasm for antiprion immunotherapy emerged when White et al. demonstrated that the passive transfer of anti-PrP IgG antibodies to wild-type mice, challenged i.p. with PrP^Sc^, resulted in a significant delay in symptoms [184]. The effects were most noticeable when a high antibody dose was administered biweekly during the splenic amplification phase. The treatment decreased splenic PrP^Sc^ at 60 days after infection, and by 250 days, PrP^Sc^ was still undetectable in the brain. An unfortunate limitation, however, was the inability, or limited ability, of the anti-PrP antibodies to cross the blood-brain barrier which restricted protection by passive vaccination to the extraneural compartments. This conclusion was inferred from results showing that passive antibody transfer had no effect on prion disease progression following intracranial challenge.

 Also in 2003, Sigurdsson et al. tested the ability of mAbs 8B4 (residues 34–52), 8H4 (175–185), and 8F9 (205–233) to provide passive protection against PrP^Sc^ challenge. Mice were inoculated intraperitoneally with scrapie at either a 10-fold or 1000-fold dilution. Beginning immediately after challenge, mice were injected weekly with one of the three mAbs. In the group receiving the 1 : 10 diluted challenge material, both 8H4 and 8B4 provided a 10% incubation prolongation, with the 1000-fold dilution, and disease prevention was observed in 10% of mice treated with 8B4. The ineffective response to 8F9 was likely due to its lower affinity for both PrP^C^ and PrP^Sc^ [185].

 The effectiveness of passive immunization appears to be restricted to the peripheral compartments as several experiments have shown development of prion disease following intracerebral inoculation despite peripheral treatment with anti-PrP antibodies. There is, however, some potential application of antibody for treatment of individuals accidentally exposed to prions. Although anti-PrP antibodies are rapidly cleared from the blood, their brief presence does not seem to stimulate any detectable autoimmune reaction. Whether this remains true for active vaccination strategies, where anti-PrP antibodies are present for much longer time periods, is a question to be addressed.

## 25. Active Immunization

Overcoming tolerance to PrP^C^ is one of the greatest challenges for active PrP immunization. Numerous investigations of different carrier systems [keyhole limpet hemocyanin [200], bacterial heatshock protein DnaK [201], and multiple antigen peptide display [188]] and adjuvants [CpG ODN [202], Freund's complete or incomplete [203, 204], Montanide [189], and TitreMax [178]] are attempts to overcome the inherent lack of immunogenicity for PrP^C^ [205]. Notably many of these potent adjuvants are not suitable for use in humans or animals due to toxicity issues [205]. Despite limitations posed by tolerance to PrP^C^, experiments have shown a relationship between antibody titers and protection from disease, therefore supporting the potential benefit of immunotherapeutic approaches.

 The first group to show a delay in prion disease onset following active immunization with recombinant PrP^C^ (residues 23–230) was Wisniewski et al. 2003. The results were modest with a 10% increase in the incubation time in mice vaccinated (s.c.) at two-week intervals starting 14 weeks prior to inoculation (i.p.). Despite a delay in symptom onset, all mice died as a result of disease progression. Those that were vaccinated at the time of inoculation did not benefit from the treatment [188]. The authors noted that high titers corresponded to prolonged incubation times, but following disease onset, there was no discernable histopathological difference among groups [188].

 Other studies, which immunized against different regions of PrP^C^, demonstrated that different epitopes of the protein may have unique therapeutic value [205]. Experiments by Schwarz et al. 2003 compared the effectiveness of vaccinating with a short synthetic peptide (residue 105–125) to recombinant mouse PrP90-230 covalently linked to keyhole limpet hemocyanin. Immunization with PrP105–125 significantly prolonged survival by an average of 23 days over the adjuvant control animals. Monoclonal antibodies with the same specificity had previously been shown to bind both PrP^C^ and PrP^Sc^. They also prevented amyloid aggregation and dissolved preformed aggregates [200, 204]. In contrast to the previous work by Wisniewski et al. with PrP23–230, the recombinant PrP90–230 immunization was not successful in delaying or preventing disease [189]. They followed up with epitope mapping experiments to determine which epitopes, within PrP90–230, produced antibodies with the highest reactivity. Antibodies to the region between 159 and 211 were the most highly detected by ELISA. The authors suggest that only antibodies specific for residues 105–125 and 144–152 were effective in preventing prion disease [189].

## 26. Mucosal Immunization

As the majority of TSEs are orally transmitted, mucosal immunization may be an effective immunotherapeutic strategy. Mucosal immunity is generally humoral and noninflammatory in nature and is therefore typically a safer alternative to subcutaneous or intramuscular immunization [206]. This may be of considerable significance as immunological issues, such as those observed in a clinical trial of an Alzheimer's vaccine (AN1792), illustrate a potential complication for the use of systemic vaccine delivery for neurological protein misfolding diseases. The AN1792 vaccine stimulates an immune response to the misfolded A*β* protein found in amyloid plaques which are thought to be responsible for causing Alzheimer's disease symptoms/pathology. The case report published by Weller et al. described a patient in the trial who rapidly declined and died six weeks following her last vaccination. Postmortem analysis revealed CD4^+^ lymphocytic meningoencephalitis accompanied by extensive macrophage infiltration of the cerebral white matter, indicative of a significant cell-mediated (Th1) inflammatory response [207]. While only one patient died, 6% of participants experienced side effects sufficiently serious to bring the trial to a premature conclusion. Experts suggested that the vaccine may have stimulated too strong an immune response which weakened the barriers that normally protect the CNS from bacterial or viral attack, or that T-cell and microglial overactivation may have induced neuroinflammation [208].

 Progress towards stimulating prion mucosal immunity was demonstrated by *in vivo* protection of mice following mucosal vaccination with PrP expressing *Salmonella typhimurium* [191]. The vaccination schedule consisted of weekly administration of four oral vaccinations using a live *Salmonella* construct followed by two injections of the killed *Salmonella* construct. Seven weeks after the first vaccination mice were exposed, via oral gavage, to 139A scrapie strain. They found that 100% of mice expressing high IgA and IgG titers, and 33% of mice with high IgG and low IgA were symptom-free 400 days after inoculation. Histological and Western blot analysis verified a lack of PrP^Sc^ in the brains of asymptomatic animals [191]. It was anticipated that the level of protection would be directly linked to the magnitude of IgA production, but the 33% survival rate observed in the low IgA/high IgG group suggested that other factors may also be important in determining vaccine efficacy [191]. They concluded that anti-PrP IgA is likely important in preventing prion uptake in the gut, while systemic anti-PrP IgG interferes with the isoform conversion process. One advantage of this system is the ability of *Salmonella* to target M cells, the key sites of prion uptake within the digestive tract [205]. Despite having only been tested in mice, the authors have provided insight into the type of immune response (noninflammatory Th2) which may be necessary for providing protection against oral prion infections. 

## 27. Immunization Using Plasmid and Viral Vectors

Tolerance represents a considerable barrier for immunotherapeutic strategies targeting PrP^C^. Despite anergy, or deletion of most B- and T-cell expressing receptors specific for PrP^C^, it may be possible to stimulate the limited anti-PrP^C^ antibody repertoire using potent immunogenic delivery systems [205, 209]. Nickles et al. hypothesized that recombinant virus-like particles (VLPs) would function as more efficient B-cell immunogens than monovalent recombinant proteins and consequently developed a retrovirus (murine leukemia virus)-based PrP vaccine construct expressing residues 121–231 (PrP^111^) [210]. Using three different groups of C57BL/6 mice with PrP genotypes (*Prnp* ^0/0, 0/+, +/+^) vaccination with PrP^111^ produced PrP^C^-specific responses with IgG titers inversely proportional to the number of PrP alleles expressed. The authors were surprised, however, by the similarity of IgM titers between the three genotypes in the early time points. While class switching of PrP^C^-specific IgM to IgG antibodies was less pronounced in wild type animals compared to *Prnp* ^0/0^, use of the murine leukemia virus delivery system appeared to provide antigenic T-helper determinants enabling class switching in the wild-type animals [210]. Whether the level of antibodies produced by this construct is sufficient to be of therapeutic value remains to be determined.

Heppner et al. engineered heterozygous prion knockout (*Prnp* ^+/o^) transgenic mice to expressed 6H4 as a single chain Ab. These mice were infected with Rocky Mountain Laboratory (RML) strain of mouse-adapted scrapie via an intraperitoneal challenge and monitored for prion disease. Mice expressing 6H4 survived 120 days longer than the control mice [186].

 In 2007, Kirnbaur's group incorporated a PrP^C^ B-cell epitope (144–152) into a capsid protein component of the bovine papillomavirus type 1 which was expressed by recombinant baculovirus technology. This carrier system is advantaged in its ability to self-assemble into VLPs which display the inserted PrP epitope at a density up to 360 copies per particle. Additionally, VLP vaccines that continuously induce anti-PrP antibody result in a more sustained antibody response over time; consequently, fewer vaccinations may be required [211, 212]. The epitope, also recognized by mAb 6H4 and other commercial antibodies, was selected based on previous studies linking that region with PrP^Sc^-induced conformational changes [211]. Rabbits and rats were vaccinated with the virus particles, along with either Freund's incomplete or CpG, four times at 2–4-week intervals. Anti-PrP IgG antibodies were detected in both species two weeks after the last boost. *In vitro* experiments using ScN2a and rabbit anti-PrP IgG demonstrated the ability of the antibodies to prevent *de novo* formation of PrP^Sc^.

Rosset et al. compared a plasmid expressing PrP as a vaccine in wild-type and *Prnp* ^0/0^ C57BL/6 mice. On its own, PrP pDNA stimulated anti-PrP antibodies directed against the N-terminal region in the knock-out mice but failed to induce a response in the wild-type animals. The addition of CpG oligodeoxynucleotides, an adjuvant that helps stimulate dendritic cells [213] and B-cells [214], to the PrP pDNA formulation helped to successfully induce anti-PrP antibodies in the wild-type mice [202]. The epitopes recognized by these antibodies were unique to those produced in *Prnp* ^0/0^ mice and specific for the C-terminal portion of PrP. Another difference was that antibodies in *Prnp* ^0/0^ mice recognized membrane-bound PrP^C^, whereas none of the antibodies from wild-type animals did. The authors suggested that antibodies were specific for different locations on PrP despite identical immunizations because B cells specific for native PrP^C^ epitopes are strongly tolerized [209]. It was also observed that *Prnp* ^0/0^ antibodies were primarily of the IgG1 isotype, whereas antibodies from wild-type mice were mainly IgG2b, an indication that the immune response in wild-type animals was skewing toward a Th1-cell-mediated response. The authors concluded that antibodies stimulated by PrP pDNA + CpG, while unable to bind PrP^C^, might be effective in blocking PrP^Sc^ replication by targeting regions uniquely exposed in the PrP^Sc^ conformation [209].

 More recently, it was suggested that the use of xenogenic antigens may be effective in circumventing tolerance to autoantigens such as PrP^C^. Although 90% of the prion amino acid sequence is conserved among mammals, it was demonstrated that vaccination of mice with bovine PrP stimulated the production of anti-PrP antibodies [215]. Tang et al. implemented xenogenic prion (human) expression via an adenovirus vector system which had been shown previously, in cancer studies, to be a potent generator of antibody (CD4^+^) and cell-mediated (CD8^+^) responses to both the viral capsid proteins as well as the transgene [216]. In C57BL/6 mice, the authors compared dendritic cell-mediated delivery with direct administration of the adenovirus vector. Only the dendritic cell mediated treatment was able to break B-cell tolerance against murine PrP, producing antibodies which increased the survival of prion-infected mice by an average of 37 days (*n* = 5) without stimulating T-cell-directed autoimmunity [190].

## 28. PrP^Sc^-Specific Immunotherapy

The understanding about the physiological function of the PrP^C^ is still limited. For this reason, it may not be the safest strategy to target an immune response towards such a commonly expressed protein. It has previously been demonstrated that antibodies which crosslink PrP^C^ in neural tissue cause apoptosis [199]. The systemic presence of autoreactive PrP^C^ antibodies may also lead to an impairment of the natural function of PrP^C^, inappropriate cell signal activation, or stimulation of suppressor T-cell lymphocytes [205]. An alternative immunotherapeutic strategy is to stimulate an immune response specific for the PrP^Sc^ conformation. While this approach is challenged by the lack of PrP^Sc^ structural information, it may present a more viable method for circumventing the body's tolerance mechanisms to PrP^C^ and avoid induction of autoimmunity.

 In 2007, Pilon et al. created three peptide vaccine constructs ranging in length from 13 to 29 aa. One construct contained a tyrosine-tyrosine-arginine motif on *α*-helix 1 which was predicted by Paramithiotis et al. to be selectively exposed on the PrP^Sc^ conformation [217]. Results from vaccination trials showed all three constructs to be moderately immunogenic with the *α*-helix 1 group displaying higher median titers than the other two groups. In a scrapie challenge experiment, the best result observed was a 20-day increase in survival when compared to the control group. There was, however, a lack of correlation between the longest surviving group and the group with the highest median titers. The authors concluded that a successful vaccine would need to overcome self-tolerance as well as stimulate antibodies with high affinity for the PrP^Sc^ conformation [218].

 One strategy for establishing an immune response to the infectious prion conformation would be to identify a cryptic epitope specifically exposed on the PrP^Sc^ conformation. In an attempt to address this issue, it was found that during the refolding process from PrP^C^ to PrP^Sc^ there is increased solvent exposure of tyrosine (Y) side chains [217]. The majority of these residues are located in the structured C-terminal domain, a region linked to infectivity and protease resistance [88]. Of the 11 residues in the structured region, 6 are present in bityrosine pairs, a motif that is conserved in human, sheep, mouse, hamster, cattle, and deer. Two pairs are in conjunction with a C-terminal arginine (R), one of which is located on *α*-helix 1 and the other on *β*-strand 2. It was hypothesized by Cashman et al. that a YY-motif located on a *β*-sheet may present an epitope specific for the PrP^Sc^ conformation. Testing of this hypothesis resulted in the production of rabbit antisera that selectively immunoprecipitated (IP) PrP^Sc^ from infected mouse brain and not PrP^C^ from uninfected brains [217]. These results indicate that the YYR epitope on *β*2-sheet may represent an appropriate vaccine target for the stimulation of PrP^Sc^-specific immune response. Despite use of an aggressive vaccination protocol which utilises the commonly used vaccine carrier molecule KLH and potent adjuvants such as Freund's complete, this strategy has been limited by poor immunogenicity where IgM was the only antibody isotype detected [217].

Using the YYR epitope as a starting point for vaccine development, our lab was able to translate this weakly immunogenic epitope into a vaccine that induces robust and consistent PrP^Sc^ immune responses in a variety of species, including sheep [219]. Specifically through dual optimization of the epitope sequence and length, as well as strategies of formulation and delivery, we generated a vaccine that induces consistent and sustained serum PrP^Sc^-specific IgG antibody responses following two vaccinations. In particular, the epitope QVYYRPVDQYSNQN, presented by a *Mannheimia haemolytica* leukotoxin carrier protein, emerges as a promising vaccine candidate. Antigen-specific antibodies were also present within cerebral spinal fluid and mucosal secretions. Furthermore, a thermodynamic algorithm has been developed to predict regions of the prion protein which are most likely to be exposed by misfolding [220]. These epitopes, as either univalent or multivalent vaccines of other PrP^Sc^-epitopes, may offer the opportunity for effective and targeted prion immunotherapy.

## 29. Peptide-Based Vaccine Design

Traditional vaccines are often designed using attenuated live or inactivated microorganisms. Culturing these organisms can be difficult, and even the safest attenuated vaccine systems can produce harmful immune responses. For diseases such as cancer, and protein misfolding diseases like TSEs, Parkinson's, Alzheimer's, and Huntington's disease, pathology is linked to altered self-proteins. As a result, normal vaccine strategies are not applicable due to robust tolerance mechanisms. Extensive research has determined that short-peptide fragments derived from residues found on the much larger self-proteins can stimulate very specific immune responses [221]. The ability to target only the “rogue” self proteins may serve as a powerful tool in preventing the transmission of these diseases or progression of symptoms/pathology in already sick individuals, without stimulating autoimmunity.

 Peptide vaccine technology offers advantages in terms of safety and ease of production. Peptide preparations can be freeze-dried making their transportation and storage much easier than those vaccines requiring refrigeration. Their safety is derived from the ability to create an immune response without the requirement for infectious material which is present in attenuated vaccines. Also, the absence of genetic material eliminates the risk of genetic integration or recombination with the patient's genome, a concern posed by DNA vaccines. Furthermore, large-scale production of peptides is economical, and purity is easily analyzed using liquid chromatography and mass spectrometry [221].

 Vaccinating with an exogenously administered peptide can be challenging because the exact pathway by which the peptide is processed and then presented to immune cells is thought to be different than for traditional antigens and not well understood [221]. To successfully activate B cells specific for a certain target, the peptide fragment has to represent the appropriate conformation. For this to be achieved, structural knowledge of the native antigen is very beneficial [222]. Consequently, peptide vaccine design for TSEs is made more challenging by the lack of structural data for the misfolded prion protein.

An additional challenge for peptide vaccine design is overcoming the weaker immunogenicity of the peptide fragments, a drawback attributed to their small size. This is particularly true of the cryptic YYR epitope identified by Cashman and Caughey as a potential peptide target for prion immunotherapy. There may be opportunity to address these limitations through the use of recombinant carrier proteins which help promote immune responses. Such systems have been shown to dramatically increase the magnitude of response against short, self-peptides [219]. Such systems may also hold cost savings advantages as the production of carrier-epitope recombinants in bacterial systems is less expensive than chemical peptide synthesis.

## 30. Challenges to a Prion Vaccine

The current priority of livestock prion diseases is CWD. CWD is the most contagious of the TSEs and presents a significant threat to both wild and farmed deer populations with rates of infectivity as high as 30% and 100%, respectively [20]. The possibility for cross-species infectivity with humans and farmed animals, as well as its ability to persist in the environment for years, and a poor understanding of CWD transmission make containment, using current resources, unattainable [7]. Efforts in regions such as Colorado suggest that culling is not an effective approach for CWD management [223]. We believe that vaccination may be an effective strategy to reduce/eliminate PrP^Sc^ shedding and to break the cycle of disease transmission. That the threat of CWD relates to both farmed and wild populations of cervids presents an additional challenge for vaccine development, while most of the strategies which have been investigated thus far have focussed on various epitopes and methods of delivery virtually all are administered through parental injection, the traditional route of vaccine delivery. While this may present an effective tool for farmed animals this is not a viable option for control of CWD in the wild.

Efficient vaccination of wildlife for control of infectious disease has been demonstrated, most notably for rabies [224]. Through this well-documented example, it is apparent that it is possible to achieve protective immunity through oral, self-administered vaccines. The implementation of rabies vaccination programs in Europe and North America has been highly successful; similar investigations have been initiated for vaccination of wild populations for Lyme disease, plague, bovine tuberculosis, and Brucellosis [225]. The release of a vaccine into the wild is not without risks and obstacles. Vaccination of wild life is only recommended as a management tool under three circumstances.

**(1)* The Disease Represents a Threat to Human Health***

There are no confirmed cases of human infection from CWD, but the zoonotic potential, through direct infection of humans or indirect infection of livestock, is of sufficient concern to be considered a threat to human health.

**(2)* The Disease Represents an Economic Threat to the Livestock Industry***

While infection of cattle with CWD has only occurred through unnatural routes of laboratory infection, it highlights the potential for these protective species barriers to be broken. Furthermore, CWD has had a devastating impact on the cervid livestock and gaming industries.

**(3)* The Disease Represents a Threat to an Endangered Species***

It is difficult to predict the long-term consequences of CWD, but modelling investigations anticipate a dramatic reduction, and potential elimination, of cervid populations [226]. From these perspectives, a wildlife vaccine for CWD is justified and appropriate.

Implementation of an oral CWD vaccine for wild cervids must also carefully consider “what success looks like.” Progression of an infectious disease in wild populations depends on natural variables (herd dynamics, rates and modes of disease transmission, and population densities). Influencing disease progression through vaccination depends on experimental variables (vaccine efficacy, vaccine mechanisms, onset and duration of immunity, timing, localization, and intensity of vaccine delivery). Similarly, the unique biology of the prion diseases, in particular the phases of peripheral amplification and CNS-associated disease pathology, may offer various opportunities to impact disease progression (Figure 2).

Following oral exposure, prion diseases progress through an initial phase of asymptomatic peripheral amplification followed by eventual transmission to the CNS. The pathology and ultimately fatal outcome of the disease is associated with accumulation of PrP^Sc^ in the brain. Peripheral amplification of prions is of importance for disease propagation within individual animals as well as transmission within populations of animals. While the precise relationship between peripheral loads of PrP^Sc^ and shedding has not been defined, it would seem intuitive that animals with lower peripheral levels of PrP^Sc^ would be less likely to shed infectious material into the environment. The carcasses of animals with lower peripheral loads of PrP^Sc^ would also be expected to contribute less to environmental contamination. Thus far the efficacy of prion vaccines has been evaluated through their ability to delay the onset of pathological symptoms, which reflects PrP^Sc^ accumulation in the CNS. However, as the vast majority of the immune response is localized to the periphery—only about 1% of circulating antibodies cross the blood-brain barrier [219]—these vaccines may have greater impact on peripheral rather than central PrP^Sc^ loads. At this time, the extent to which the transition of the disease from periphery to CNS reflects peripheral loads of PrP^Sc^ or a temporal relationship which is independent of peripheral PrP^Sc^ quantities has not been defined. Theoretically, a vaccine could have significant impact on peripheral PrP^Sc^ while having only minimal impact on the timing and severity of disease pathology. While offering minimal benefit to an individual animal, such a vaccine may be valuable from a population perspective by reducing shedding and minimize disease propagation.

## 31. Importance of a TSE Vaccine

As a group, TSEs have had undeniable economical impact, leaving many countries with billions of dollars in lost revenue due to trade embargos and weakened consumer confidence. While key historical TSE events have provided the momentum for research initiatives aimed at understanding PrP^C^ function, PrP^Sc^ infectivity, and designing effective premortem diagnostics and therapeutic strategies, definitive answers in these areas remain elusive. Despite the research initiatives that have been underway for decades, there are still large deficits in key areas of understanding such as PrP^C^ function and pre-mortem diagnostics. Identifying a disease specific epitope may have significant applications in more than one of these TSE research areas. These events have provided the momentum for research initiatives aimed at understanding PrP^C^ function, PrP^Sc^ infectivity, and designing effective diagnostic tests and therapeutic strategies.

## Figures and Tables

**Table 1:** Comparative analysis of prion diseases of food animals.

TSE	Age of onset (yr)	Disease variants	Mode of transmission	Genetic predisposition	Clinical signs	Cross-species infectivity
Scrapie	2–4 [33]	Classical atypical/Nor98	Amniotic fluid and placenta [34]	Susceptibility 136V, 154R, 171Q [15] Reduced susceptibility 136A, 154R, 171R [16]	Weight loss Ataxia Abnormal behavior Sensory changes Incoordination Head tremors Recumbency [35]	Cattle, goat, mouse, hamster, rat, bank vole, deer, and elk [36–38]

BSE	4-5 [39]	Classical H-type L-type	Contaminated feed [39]	Susceptibility [40, 41] 23 bp *PRNP* promoter insertion/deletion 12 bp *PRNP* intron insertion/deletion E211K	Weight loss Ataxia Abnormal behavior Sensory changes Incoordination Bradycardia Reduced rumination Decreased milk yield [42]	Bison, sheep, goat, deer, pig, mink, marmoset, cat, mouse, human, nonhuman primates [43]

CWD	2–4 [24]	Possible varied conformer population [44, 45]	Animal contact and environmental contamination [20]	Susceptibility [20] Elk-132MM Mule Deer-132SS–225SS Cervid rigid Loop-S170N [46] -N174T [47] Reduced susceptibility [48] White tail Deer-Q95H-G96S	Weight loss Abnormal behavior Listlessness Incoordination Polydipsia Polyuria Hypersalivation Lowering of the head and ears Anorexia [21]	Moose, cattle, sheep, goat, mouse, hamster, ferret, mink, and squirrel monkey [20]

**Table 2:** Comparative analysis of *in vitro* and *ex vivo* immunotherapeutic investigations of prion diseases.

	Region	Model	Efficacy	Reference
	mAb 3F4 (109–113) pSera; 23–33, 90–104, 143–156, 219–232	Cell-free conversion	pSera 219–232 disrupted formation of new PrP^Sc^	Gilch et al. 2003 [178]
	mAb 6H4 (144–152)	ScN2a cell line	Clearance of PrP^Sc^ from cell line following antibody treatment	Enari et al. 2001 [177]
	Fab D18 (132–156)	ScN2a cell line	Blocked PrP^Sc^ formation and cleared existing PrP^Sc^	Peretz et al. 2001 [179]
*In vitro*	SAF34 (octarepeat) SAF61 (144–152)	ScN2a cell line overexpressing PrP^C^	Both Abs decreased PrP^Sc^ and PrP^C^ in infected and uninfected cells	Perrier et al. 2004 [180]
	Panel of antibodies to PrP^C^ and PrP^Sc^ generated in PrP^0/0^ mice	ScN2a cell line	Capacity for protection independent of epitope but dependent on ability to bind cell surface PrP^C^ to prevent internalization	Kim et al. 2004 [181]
	ER targeted scFv of 8H4 (175–185) and 8F9 (225–231)	Expression in PC12 cell line	Inhibition of PrP^C^ translocation to cell surface; prevented PrP^Sc^ accumulation	Cardinale et al. 2005 [182]
	mAbs to “YYR” motif	ScN2a cell line	Reduced content of PrP^Sc^	Cashman and Caughey 2004 [168]

*Ex vivo*	PrP^C^ poly Ab *ex vivo* neutralization	Golden hamster	2-log reduction	Gabizon et al. 1988 [174]
	Fab D18 (132–156) *Ex vivo* neutralization	ScN2a cell line	Mice lived 3 months longer (3-log reduction of infectivity)	Peretz et al. 2001 [179]

**Table 3:** Comparative analysis of *in vivo* immunotherapeutic investigations of prion diseases.

* In vivo *				
Treatment	Region	Model	Efficacy	Reference
Passive	scFv 8H4 (145–180)	Lysates from scFv PC12 cells i.c. injected into mice 35 days after scrapie exposure	2/10 mice protected from 8/10 infection mice symptom-free after 300 days	Vetrugno et al. 2005 [183]

Passive	Anti-PrP IgG Abs ICSM 35 (94–105) ICSM 18 (144–152)	Mice challenged i.p. with scrapie, Ab treatment twice weekly	Signficant delay in prion symptoms. Reduction in splenic PrP^sc^ and delayed transfer to brain	White et al. 2003 [184]

Passive	mAb 8B4 (34–52) mAb 8H4 (175–185) 8F9 (205–233)	Mice challenged i.p. with scrapie, weekly treatment with Ab	8H4 and 8B4 10% longer incubation period at high challenge dose. At low challenge dose, 8B4 prevented disease in 10% of animals	Sigurdsson et al. 2003 [185]

Passive (engineered)	Transgenic mice expressing 6H4 (144–152) as single chain-Ab	Mice challenged i.p. with scrapie	Prolonged survival by 120 days	Heppner et al. 2001 [186]

Passive (engineered)	PrP^C^ specific scFv	Expression specifically in CNS with recombinant adenoassociated vector type 2 viral vector platform	Delayed onset in peripherally inoculated mice	Wuertzer et al. 2008 [187]

Active	rPrP^C^ 23–230	s.c. vaccination of mice	10% increase in incubation time; correlated with Ab titres	Sigurdsson et al. 2003 [188]

Active	Peptide 105–125 rPrP^C^ 90–230	Mice immunized and orally challenged with infected brain homogenate	Peptide improved survival by 23 days; protein had no effect	Schwarz et al. 2003 [189]

Active (engineered)	Human PrP^C^	Transfer of adenotransduced dendritic cells. Mice challenged i.p.	Prolonged survival times	Rosset et al. 2009 [190]

Active (mucosal)	Salmonella expressing PrP	Four oral vaccinations with live salmonella; 2 with dead Challenge via oral lavage	100% of mice expressing high IgA and IgG and 33% of mice with high-IgG and low-IgA symptom-free after 400 days	Goñi et al. 2008 [191]
